# Effect of metal-containing topical agents on surface doses received during external irradiation

**DOI:** 10.1093/jrr/rry078

**Published:** 2018-09-22

**Authors:** Ayumi Iyama, Tomohiko Matsuyama, Eriko Matsumoto, Takafumi Araki, Satoshi Inokuchi, Mizuki Yamashita, Noritoshi Honda, Taiga Miyake, Tetsuo Saito, Ryo Toya, Yudai Kai, Yasuyuki Yamashita, Natsuo Oya

**Affiliations:** 1 Department of Radiology, Kumamoto University Hospital, 1-1-1, Honjo, Kumamoto, Kumamoto, Japan; 2 Department of Radiation Oncology, Kumamoto University Hospital, 1-1-1, Honjo, Kumamoto, Kumamoto, Japan; 3 Department of Radiology, Amakusa Central General Hospital, Kumamoto, Japan; 4 Department of Dermatology, Amakusa Central General Hospital, Kumamoto, Japan

**Keywords:** radiation dermatitis, topical agent, surface dose, zinc oxide, silver sulfadiazine

## Abstract

The ability of topical metal-containing agents (MCAs) to enhance radiation dermatitis remains controversial. In the present study, we evaluated increases in surface doses associated with topical agents at different application thicknesses and with MCAs versus non-metal containing agents (NMCAs). We assessed two clinically available MCAs, zinc oxide ointment (ZOO) and silver sulfadiazine cream (SSDC), and eight NMCAs. Surface doses were measured using a Markus chamber placed on a polystyrene phantom. To evaluate the role of application thickness, each agent was applied to the chamber in oil-slick (<0.1-mm), 1-mm and 5-mm layers prior to irradiation of a 10 × 10 cm field with 4-, 6- and 10-MV X-ray beams. The surface dose enhancement ratio (SDER) was calculated as the ratio of the surface dose with an agent to the dose without an agent. The SDER values for the eight NMCAs, ZOO and SSDC at an oil-slick thickness were 101.6–104.6% (mean: 103.3%), 104.5% and 105.0%, respectively, using a 6-MV X-ray beam. The corresponding values at a 1-mm thickness were 196.8–237.8% (mean: 215.7%), 229.3% and 201.4%, respectively, and those at a 5-mm thickness were 342.2–382.4% (mean: 357.9%), 357.1% and 352.6%, respectively. A similar tendency was found using 4- and 10-MV X-ray beams. The lack of a significant difference in surface dose enhancement between MCAs and NMCAs, particularly when applied in oil-slick layers, suggests that MCAs do not need to be avoided or applied in a restricted manner during radiotherapy for dosimetric reasons.

## INTRODUCTION

Radiation dermatitis is one of the most common side effects of radiation therapy. Despite the publication of clinical guidelines for skin care management during radiation therapy [1–3], sufficient evidence of the ability of the recommended measures to effectively prevent and manage radiodermatitis has not yet been provided. Recent progress in radiation therapy technology has led to a decreased incidence of severe radiation dermatitis. However, some patients still develop dermatitis with sores and bleeding when radiotherapy is administered to the head and neck, vulva, and/or in combination with chemotherapy or molecular targeted therapy, and these patients occasionally find it difficult to continue therapy.

Clinicians have often been reluctant to use metal-containing agents (MCAs) during radiotherapy, given the risk of interaction of these agents with radiation [4–6]. To our knowledge, however, no clinical trial has examined whether the use of MCAs worsens dermatitis, and some reports have even suggested the usefulness of MCAs [e.g. silver sulfadiazine cream (SSDC) [1, 3, 7, 8] and zinc oxide cream (ZOC) [8]] for radiation dermatitis. Therefore, an investigation of the dermal effects of MCAs during radiotherapy is needed.

Two previous dosimetry studies evaluated the potential effects of MCAs on surface radiation doses [9, 10]. First, Burch *et al.* reported no significant differences in surface doses associated with normally applied MCAs and non-metal-containing agents (NMCAs) during irradiation with a 6-MV X-ray beam [9]. Second, Fackrell *et al.* compared the dose effects of two MCAs (SSDC and ZOC) and one NMCA [aqueous cream (AQC)] during irradiation with a 6-MV X-ray beam, and reported that a slightly lower dose was required with ZOC than with AQC or SSDC [10]. However, neither study strictly addressed the possibility that slight differences in application thickness might affect the surface dose in the build-up region [9, 10]. Furthermore, Burch *et al.* evaluated clinically impractical topical agents [9]. Conclusions regarding the use of MCAs during radiotherapy in clinical practice should be made using dosimetric data associated with clinically available agents and a fixed application thickness. Accordingly, the present study aimed to examine specifically whether the presence of a metallic element would affect the surface dose, using clinically available MCAs and NMCAs at consistent application thicknesses.

## MATERIALS AND METHODS

We measured the surface doses associated with 10 clinically available topical agents (i.e. creams and ointments), including two MCAs [zinc oxide ointment (ZOO) and SSDC] and eight NMCAs. ZOO contains 20% (200 mg/g) zinc oxide, and SSDC contains 1% (10 mg/g) silver sulfadiazine. We note that one of the NMCAs, a mixture of sugar and povidone-iodine, contains 3% (30 mg/g) povidone-iodine, which, although non-metallic, has a relatively high atomic number. The physical properties of all agents are listed in Table 1.

**Table 1.** Comparison of the physical properties of 10 topical agents

No.	Generic name	Active ingredient (mass percentage)	Molecular formula	Brand name	Specific gravity
1	White petrolatum	White petrolatum (100%)	C_15_H_32_ to C_20_H_42_	PROPETO®	0.815–0.880
2	Dimethyl isopropyl azulene ointment	Dimethyl isopropylazulene (0.033%)	C_15_H_18_	Azunol Ointment 0.033%	0.870
3	Betamethasone valerate ointment	Betamethasone valerate (0.12%)	C_27_H_37_FO_6_	Rinderon®-V	0.870
4	Lysozyme hydrochloride ointment	Lysozyme hydrochloride (5%)	C_616_H_963_N_193_O_182_S_10_·*x*HCL	REFLAP® Ointment 5%	0.940
5	Heparinoid ointment	Heparinoid (0.3%)	Not applicable	Hirudoid® Soft Ointment	1.000
6	Diphenhydramine cream	Diphenhydramine (1%)	C_17_H_21_NO	RESTAMIN KOWA CREAM 1%	1.013–1.020
7	Macrogol ointment	Macrogol 400 (50%)	HOCH_2_(CH_2_OCH_2_)_n_CH_2_OH^b^	SOLBASE ®	1.110–1.140
		Macrogol 4000 (50%)	*n* = 7–9 *n* = 59–84		
8	Mixture of sugar and povidone-iodine	Povidone-iodine (3%)	(C_6_H_9_NO)_*n*_·*x*I	U-PASTA KOWA Ointment	1.270
		Sucrose (70%)	C_12_H_22_O_11_		
9	Zinc oxide ointment^a^	Zinc oxide (20%)	ZnO	Zinc Oxide Ointment ‘Hoei’	1.064
10	Silver sulfadiazine cream^a^	Sulfadiazine silver (1%)	C_10_H_9_AgN_4_O_2_S	GEBEN cream 1%	0.980–0.990

^a^Zinc oxide ointment (No. 9) and silver sulfadiazine cream (No. 10) contain metallic elements. The other agents do not contain metallic elements.^b^*n* ranging from 7 to 9 in Macrogol 400, and from 59 to 84 in Macrogol 4000.

**Fig. 1.**
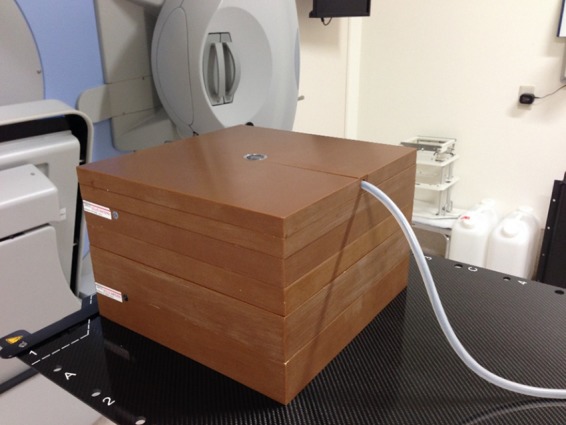
A Markus chamber set in a solid water phantom.

**Fig. 2.**
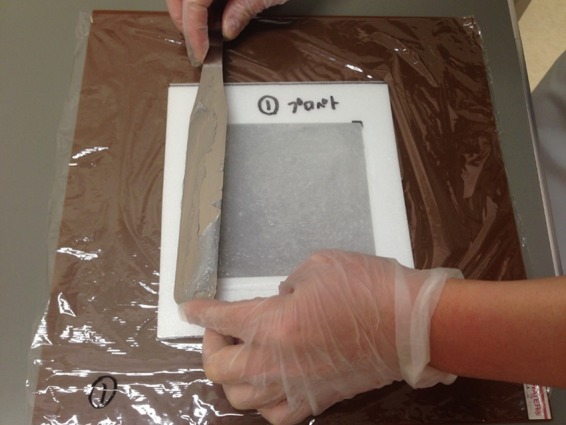
Specific frames were used to apply 1-mm thick and 5-mm thick layers to a thin polyethylene sheet.

Surface doses were measured using a Markus-type parallel plate ionization chamber (PTW, Freiburg, Germany) placed on a polystyrene phantom and an electrometer (RAMTEC Smart, Toyo Medic Co., Ltd, Tokyo, Japan) (Fig. 1). Although a previous report noted that this chamber overestimated surface doses in build-up regions [11], we did not correct for this effect because the comparisons made in this study were relative. For the experiment, each agent was applied to a thin polyethylene sheet (<0.1 mm) positioned over the chamber window, at the following thicknesses: oil-slick thickness, which is <0.1 mm, using the finger-tip unit [12]; 1 mm, which was selected as a realistic amount to apply using medical gauze; and 5 mm, which is rarely used in practice but was selected for dosimetric comparison. For the latter two thicknesses, uniform layers were created by filling an 11 × 11 × 0.1 cm or 11 × 11 × 0.5 cm well, respectively, in a polystyrene frame and smoothing the surface as much as possible (Fig. 2).

**Fig. 3.**
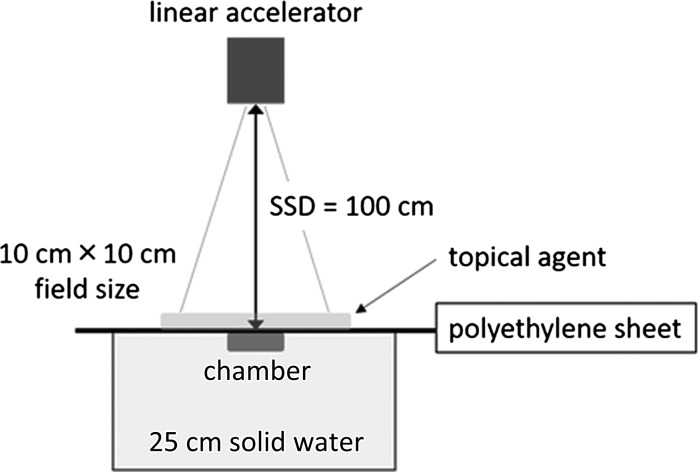
Diagram of a phantom with a Markus-type chamber. SSD, source-to-surface distance.

The chamber window surface was set at a source-to-surface distance of 100 cm (Fig. 3). Each agent was irradiated with 100 monitor units under 4-, 6- and 10-MV photon beams in a field size of 10 × 10 cm, using an Elekta Synergy linear accelerator (Elekta Instrument AB, Stockholm, Sweden). Surface doses were measured using the protocol published by Task Group 51 of the Radiation Therapy Committee of the American Association of Physicists in Medicine [13]. Each agent was subjected to irradiation three times. The uncertainty of the three measurements was negligible (i.e. the coefficient of variation was ≤0.40%), and the mean value of the surface doses of three measurements was used for the calculations. We defined the surface dose enhancement ratio (SDER) for each topical agent at each thickness as follows: (surface dose with an agent)/(surface dose without an agent) × 100 (%).

## RESULTS

**Fig. 4.**
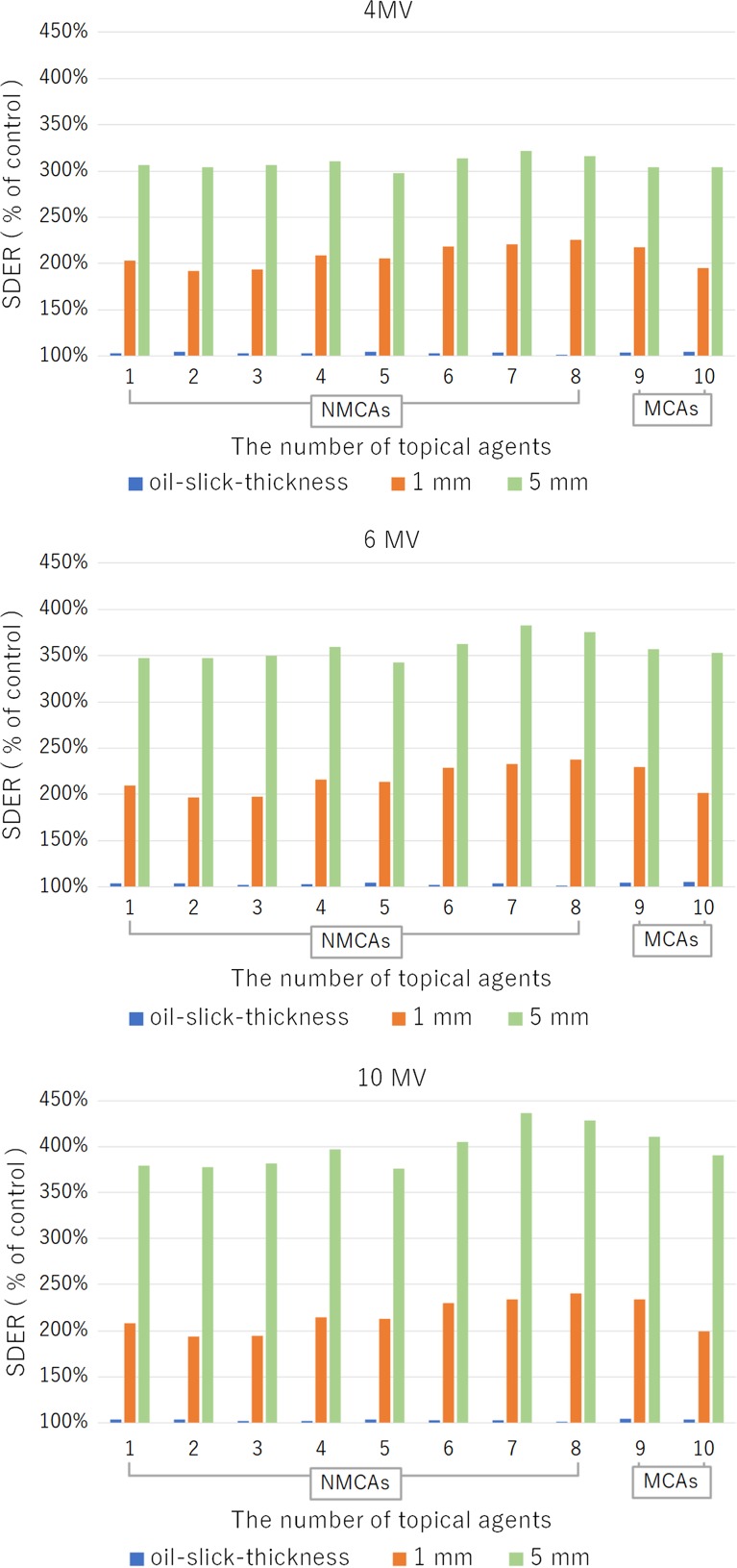
The surface dose enhancement ratio (SDER), or the ratio of the surface dose with an agent to the dose without an agent (i.e. control) was calculated for each agent at oil-slick, 1-mm and 5-mm thicknesses using 4- (a), 6- (b), and 10-MV (c) photon beams. The surface dose without an agent (i.e. only a polyethylene sheet placed over the chamber) was used as the control. The *x*-axis indicates the 10 topical agents by number (see Table 2 for further details).

Figure 4 and Table 2 present the SDER associated with each topical agent. When using a 6-MV X-ray beam, the SDER values for the eight NMCAs, ZOO and SSDC were 101.6–104.6% (mean: 103.3%), 104.5% and 105.0%, respectively, at an oil-slick thickness. The corresponding values were 196.8–237.8% (mean: 215.7%), 229.3% and 201.4%, respectively, at a 1-mm thickness and 342.2–382.4% (mean: 357.9%), 357.1% and 352.6%, respectively, at a 5-mm thickness. A similar tendency was found when using 4- and 10-MV X-ray beams (Fig. 4, Table 2).

**Table 2.** The surface dose enhancement ratio (SDER) for 10 topical agents at the indicated thicknesses

Topical agents	SDER (%)								
	Oil-slick thickness			1-mm thickness			5-mm thickness		
	4 MV	6 MV	10 MV	4 MV	6 MV	10 MV	4 MV	6 MV	10 MV
NMCAs^a^									
1. White petrolatum	103.4	103.8	103.2	203.6	209.9	207.9	306.6	347.2	379.9
2. Dimethyl isopropyl azulene ointment	104.3	104.1	103.3	192.2	196.8	193.4	304.4	347.3	378.0
3. Betamethasone valerate ointment	102.7	102.4	101.9	193.4	197.9	194.1	306.3	349.7	381.7
4. Lysozyme hydrochloride ointment	102.9	102.8	102.3	208.6	216.3	214.8	310.5	359.2	397.6
5. Heparinoid ointment	104.8	104.6	103.4	205.8	213.7	212.8	297.9	342.2	376.3
6. Diphenhydramine cream	102.7	102.6	102.7	218.6	228.9	230.2	313.7	362.4	405.6
7. Macrogol ointment	103.7	103.5	103.0	221.1	232.8	234.0	322.0	382.4	436.5
8. Mixture of sugar and povidone-iodine	101.7	101.6	100.8	225.4	237.8	240.6	316.3	375.0	428.5
MCAs^a^									
9. Zinc oxide ointment	104.2	104.5	104.3	217.5	229.3	234.2	303.8	357.1	411.0
10. Silver sulfadiazine cream	104.9	105.0	103.9	195.4	201.4	199.1	304.3	352.6	391.1

^a^MCA, metal containing agent, NMCA, non-metal containing agent.

## DISCUSSION

We observed no significant differences in surface dose enhancement between MCAs and NMCAs at any application thickness, but particularly at an oil-slick thickness (<0.1 mm) using 4-, 6- and 10-MV X-ray beams. We note that this result is highly significant, as an oil-slick thickness is generally applied in clinical practice, using the finger-tip unit [12]. We further note that although we observed slight variations in surface doses at 1-mm and 5-mm thicknesses, these variations did not always correspond to higher values with MCAs. The values obtained with SSDC, a cream containing only 1% metallic component (sulfadiazine silver), were nearly the same as or lower than those obtained with most NMCAs. Furthermore, although ZOO has a relatively high metallic concentration (20% zinc oxide), NMCAs with higher specific gravities [macrogol ointment (No. 7) and mixture of sugar and povidone-iodine (No. 8)] yielded higher surface dose increases. This correlation of dose changes with specific gravity is reasonable, as Compton scattering is the main reciprocal action associated with the high-energy X-rays used for radiotherapy [14]. Although clinicians have generally tended to restrict the use of MCAs during radiotherapy to avoid possible interactions with radiation, our results suggest that, physically, this caution is unwarranted.

The minor effects of clinically available MCAs on dosimetry may be attributable to the relatively low concentrations of metallic elements in these agents. It is generally recognized that there is a complex relationship of bolus effect, scattering, and absorption with surface dose changes associated with topical agents. A previous study of dental materials suggested that the effects of scattering and absorption during high-energy X-ray irradiation increased with the inclusion of higher density or higher atomic number substances in the irradiation field [15]. However, topical agents contain much lower concentrations of metallic elements, compared with dental materials, leading us to conclude that these agents had little effect on scattering and absorption in our study. In addition, our study suggested that the application thickness could cause a bolus effect and would have a stronger influence on the surface dose than the type of agent. However, we should note that a previous Markus-type chamber-based analysis overestimated surface doses in build-up lesions [11].

Although previous studies have also evaluated the influences of MCAs on surface doses [9, 10], we note the significance of our study, which used only clinically available topical agents and strictly determined application thicknesses. In a previous analysis, Burch *et al.* investigated surface dose changes associated with 15 products, both with and without high atomic number components [9], and did not detect any large increases in surface dose with normal product application. Although this result is similar to our findings, the earlier study evaluated only deodorants, powders and lotions, rather than practical topical agents (i.e. creams and ointments), and the products were not applied in specific thicknesses. In a second study, Fackrell *et al.* compared the dose effects of two MCAs (SSDC and ZOC) and one NMCA (AQC) [10], and observed no significant differences in dose effect between SSDC and AQC, and a slight decrease in dose when using ZOC. Although the authors used identical weights of the three agents, the agents were not applied at identical thicknesses because the authors did not consider specific gravities. Therefore, their measurements may have been insufficiently accurate, as a slight difference in thickness might affect the surface dose in a build-up region. We conclude that, when measuring surface doses, it is important to strictly determine the application thickness and thus exclude differences due to the bolus effect while evaluating the influences of the components in various agents.

Our results may increase the clinical selection of topical agents available for patients with radiation dermatitis. Both ZOO [16] and SSDC [17] are known to be beneficial for dermatitis therapy, and, in particular, zinc can promote the healing of skin and mucous membrane wounds [18]. Accordingly, ZOO is very suitable for mild dermatitis, and its early use may reduce and even prevent radiation dermatitis. Although a previous report described the use of ZOO as a treatment for Grade 2 and 3 radiation dermatitis in patients with squamous cell carcinoma of the head and neck [8], no randomized controlled trial (RCT) has demonstrated the utility of ZOO for this purpose, possibly because of concerns regarding interactions of the metallic components of ZOO with radiation. However, our study found no dosimetric reason to restrict the use of ZOO during radiotherapy, and we consider this agent to be useful in this context. Future clinical studies regarding the use of ZOO for radiation dermatitis are expected in the future. We note that several reports have also described SSDC as a useful therapeutic agent for radiation dermatitis [1, 3, 7, 8].

We must note two limitations of our study. First, we only evaluated dose changes in the vertical beam, and not in the oblique or tangent beam. However, a recent Monte Carlo simulation-based study addressed this limitation [19], and showed that a product thickness of <0.7 mm was not likely to cause a clinically meaningful dose increase in a 10 × 10 cm field across a range of gantry angles (0°–80°). No significant bolus effects associated with increased skin doses were observed in that study, particularly at a thickness of <0.1 mm (i.e. ‘oil-slick thickness’ in our study) [19]. Considering these results and our results, we suggest that no topical agent would be problematic when applied clinically at an oil-slick thickness. Second, we did not evaluate actual patients, and therefore could not consider factors such as the effect of skin temperature on agent properties, or the skin permeability, or the pharmacological effects of each agent. However, several previous RCTs demonstrated that the use of aluminum-containing antiperspirants or deodorants did not affect skin reactions during adjuvant breast radiotherapy [20, 21].

In conclusion, we observed no significant differences in surface dose enhancement between MCAs and NMCAs at any thickness, especially at the oil-slick thickness used clinically. Therefore, dosimetric restrictions on the use of MCAs during radiotherapy may be unnecessary. Although an RCT of clinically available topical agents remains to be conducted, our physical study indicated no significant influence of metallic components in topical agents.
